# Practical real-time MEG-based neural interfacing with optically pumped magnetometers

**DOI:** 10.1186/s12915-021-01073-6

**Published:** 2021-08-10

**Authors:** Benjamin Wittevrongel, Niall Holmes, Elena Boto, Ryan Hill, Molly Rea, Arno Libert, Elvira Khachatryan, Marc M. Van Hulle, Richard Bowtell, Matthew J. Brookes

**Affiliations:** 1 Laboratory for Neuro- and Psychophysiology, Department of Neurosciences, KU Leuven, Leuven, Belgium; 2 Leuven Institute for Artificial Intelligence (Leuven.AI), Leuven, Belgium; 3 Leuven Brain Institute (LBI), Leuven, Belgium; 4 Sir Peter Mansfield Imaging Centre, School of Physics and Astronomy, University of Nottingham, Nottingham, UK

**Keywords:** Brain-computer interface (BCI), Optically pumped magnetometers (OPM), Event-related potential (ERP), Event-related field (ERF), Steady-state visual evoked potential (SSVEP), Magnetoencephalography (MEG), Electroencephalography (EEG)

## Abstract

**Background:**

Brain-computer interfaces decode intentions directly from the human brain with the aim to restore lost functionality, control external devices or augment daily experiences. To combine optimal performance with wide applicability, high-quality brain signals should be captured non-invasively. Magnetoencephalography (MEG) is a potent candidate but currently requires costly and confining recording hardware. The recently developed optically pumped magnetometers (OPMs) promise to overcome this limitation, but are currently untested in the context of neural interfacing.

**Results:**

In this work, we show that OPM-MEG allows robust single-trial analysis which we exploited in a real-time ‘mind-spelling’ application yielding an average accuracy of 97.7%.

**Conclusions:**

This shows that OPM-MEG can be used to exploit neuro-magnetic brain responses in a practical and flexible manner, and opens up new avenues for a wide range of new neural interface applications in the future.

**Supplementary Information:**

The online version contains supplementary material available at (10.1186/s12915-021-01073-6).

## Background

It is widely believed that technologies will interact directly with our brains in the future [1], enabling the restoration [2–4] or augmentation [5] of neural functionality. A crucial aspect for the success of these neurotechnologies is the ability to reliably capture high-quality measurements of cortical activity. Invasive neural recordings from electrocorticography (ECoG) [6], micro-electrode arrays [7] or depth electrodes [8] have reported the most promising demonstrations (e.g. direct prosthetic control [9] and decoding of speech [10, 11]). However, due to the invasive nature of the brain implants used, these can only be considered for chronic cases (e.g. ALS [6] or tetraplegics [12]) and for applications that serve continuous (i.e., daily) use (e.g. communication [13] and control [14]). These restrictions inherently limit the targeted end-users and applications. Similar electrical signals can also be recorded non-invasively from the scalp using scalp mounted electroencephalography (scalp-EEG). While this opens up neurotechnologies for a larger population and applications (e.g. neurorehabilitation therapies [15] or games [16, 17]), the scalp-recorded potentials are heavily distorted by the conduction through the cerebrospinal fluid, skull and skin layers which results in a lower information content.

Despite often being overlooked, the complementary activations in the magnetic domain are considerably less distorted by the inhomogeneous conductivity of the head. Consequently, magnetoencephalography (MEG) provides information at a higher spatial resolution [18] compared to scalp-EEG. However, recording MEG is not trivial as the traditional acquisition hardware is based on superconducting quantum interference devices (SQUIDs) that require constant cryogenic cooling, making it an expensive and restrictive technique. The device is also optimised for adult subjects and is highly sensitive to head movement artefacts [19], making it impractical for deployment in the context of Brain-Computer Interfacing (BCI) or for patients suffering from motor dysfunction.

Optically pumped magnetometers (OPMs) are a promising new technology for MEG that overcomes the practical drawbacks of SQUID-based systems. OPMs are small, lightweight sensors that are sensitive to small changes in magnetic field [20]. Because they operate without the need for coolants or external thermal regulators [21], OPM sensors can be placed in contact with the skin at any location [22, 23], leading to an improved sensitivity compared to traditional MEG [24] and opening new avenues of neuro-magnetic research previously deemed impossible [25, 26]. While initial reports of OPM-technology present promising results [22, 27], it is currently unclear whether this technology provides sufficient signal quality and/or stability for the (real-time) single-trial analysis/decoding that is required in the context of a BCI application.

In this study, we investigated and analysed the neural responses using two BCI stimulation paradigms, one based on event-related potentials/fields (ERP/ERF) and another on steady-state visual evoked potentials (SSVEP). We developed a real-time proof-of-concept that allows the user to spell text without the need for efferent pathways (e.g. muscular control). We demonstrate that the proof-of-concept speller enables reliable communication, thereby establishing OPM-MEG as a new recording technology for non-invasive BCIs.

## Results

### ERP/ERF-based BCI

**Fig. 1.**
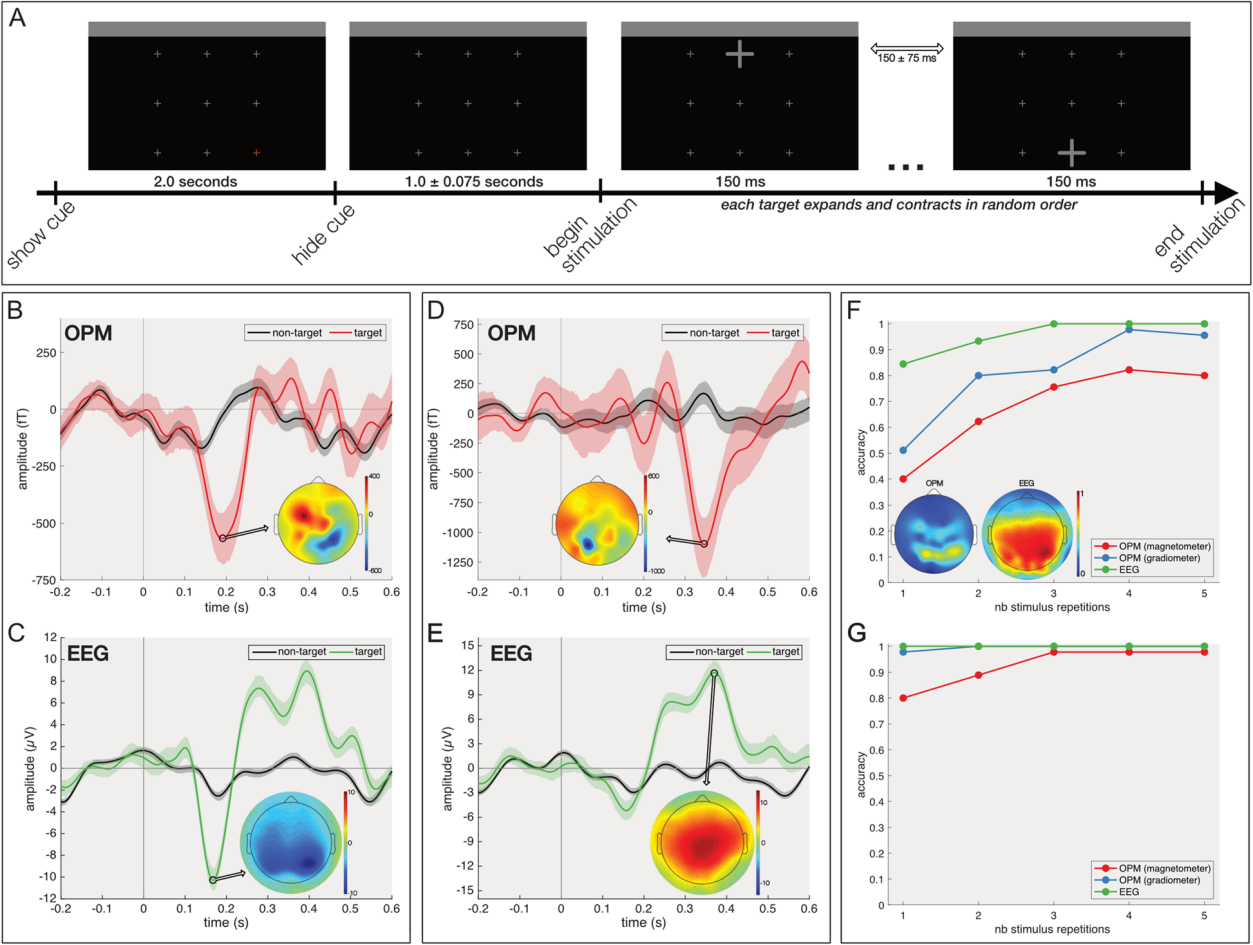
Motion-onset visual evoked potential. **a** A schematic showing one trial of the experimental paradigm. **b** ERF (filtered between 0.5 and 15 Hz) at the OPM channel exhibiting the largest M200 amplitude. The embedded scalp plot shows the spatial activation pattern when the maximal M200 amplitude is reached. The full line indicates the average ERF and the shaded area the 95% confidence interval. **c** ERP (filtered between 0.5 and 15 Hz) at the scalp-EEG channel having the largest N200 amplitude. The embedded scalp plot shows the spatial activation pattern when the maximal N200 amplitude is reached. The full line indicates the average ERP and the shaded area the 95% confidence interval. **d** M300 ERF (similar conventions as in **b**). **e** P300 ERP (similar conventions as in **c**). **f** Accuracy of decoding the gazed target at the best OPM sensor, scalp-EEG electrode and OPM gradiometer for increasing stimulus repetitions. Embedded scalp plots show the average decoding accuracy across the scalp. **g** Multi-channel decoding accuracy for scalp-EEG and OPM for an increasing number of stimulus repetitions


One of the first BCIs described in literature exploited neural responses that are time-locked to the onset of a visual stimulus. These so-called event-related potentials (ERPs), or event-related fields (ERFs) as they are termed in MEG, have since been widely adopted for BCI purposes. In a first experiment, we aimed at investigating the feasibility of developing an OPM-based BCI that employs these ERPs/ERFs. To this end, we developed an interface presenting nine crosses (Fig. 1a, Additional file 1), each one spanning a visual angle of 0.86^∘^. One subject (male, aged 28 years) completed 45 trials, in each of which he was asked to attend a cued cross. During a trial, each of the nine crosses were expanding to a visual angle of 4.3^∘^ and contracting back to their original size in a timespan of 150 ms with a jittered inter-stimulus interval of 150 ±75 ms. To contrast the OPM responses with traditional scalp-EEG, the same participant completed this experiment twice, once with each recording modality. Whenever the gazed cross expands, a motion-onset visual evoked potential (mVEP) is expected in the obtained ERP/F. In scalp-EEG, the mVEP is expected as a strong negative deflection around 200 ms post-stimulus onset, which we will refer to as the N200. As the magnetic component is tangential to the neural dipole, the polarity of the obtained ERF is not directly related to the polarity of the neural dipole as measured by EEG. Following the conventions in the MEG literature, we will refer to the ERF elicited by the motion-onset paradigm as M200.

Additional file 1: Video showing one trial of the stimulation paradigm during the motion-onset experiment


Both the OPM and scalp-EEG recordings exhibit a clear N/M200 component which reaches its maximal amplitude over the parieto-occipital scalp area (Fig. 1b,c, Additional files 2 and 3). The maximal amplitude of (-)567.34 fT and − 10.30 *μ*V was reached at a latency of 191 ms (95% confidence interval between 179 and 205 ms) and 168 ms (95% confidence interval between 166 and 171 ms), respectively. Note that the difference is latency might be partially attributed to inter-session variability as the data from the two recording modalities were obtained in different sessions. A signal-to-noise (SNR) analysis comparing the N/M200 amplitudes with respect to their (pre-onset) baseline amplitude (Additional file 4) reveals that the SNR of the M200 ERF (maximal SNR of 20.48 dB, 95% confidence interval between 16.27 and 25.58 dB) is similar the one of the N200 ERP (maximal SNR of 18.11 dB, 95% confidence interval between 13.68 and 23.23 dB). Given that both SNR confidence intervals largely overlap, this difference is not deemed significant. In addition to the single-magnetometer case, we also investigated the use of OPM pairs to form planar gradiometer channels. While these are similar to bipolar channels used in scalp-EEG studies, unlike the latter, the OPM gradiometers are able to take advantage of the polarity reversal at opposite sides of the neural dipole to boost the SNR. Indeed, the gradiometer channel exhibiting the highest SNR among all possible OPM-pairs reaches 22.29 dB (95% confidence interval between 16.72 and 28.18 dB).


Additional file 2: Video showing the spatial topography of the average ERF when the gazed target is moving.



Additional file 3: Video showing the spatial topography of the average ERP when the gazed target is moving.


In addition to the visual N/M200 component, our stimulation paradigm also elicits a cognitive P/M300 component over the centro-parietal scalp area (Fig. 1d, e, Additional files 2 and 3). The maximal amplitude of (-)1103.38 fT and 11.71 *μ*V of this second component is reached at a latency of 347 ms (95% confidence interval between 343 and 351 ms) and 370 ms (95% confidence interval between 363 and 376 ms), for OPM-MEG and scalp-EEG respectively. Similar to before, an SNR analysis of the P/M300 component (Additional file 4) reveals similar SNR values for both recording modalities. Among the OPM magnetometers, a maximal SNR of 18.64 dB (95% confidence interval between 12.39 and 25.18 dB) is found, while for scalp-EEG, the maximal SNR is 22.04 dB (95% confidence interval between 18.86 and 26.16 dB). The OPM gradiometer channel with maximal SNR has a value of 21.48 dB (95% confidence interval between 15.92 and 28.47 dB). The largely overlapping confidence intervals suggest that the ERP/F components of both recording modalities do not significantly differ in terms of SNR.

As the N/M200 component is only present when the gazed cross is expanding, a decoding model can be used to identify the gazed target from the brain’s responses. In brief, by allowing all nine crosses to expand a number of times and obtaining the average ERP/F per cross, the classification model will identify which ERP/F contains the N/M200 component, allowing us to detect the gazed cross. Note that the accuracies described below are obtained using a cross-validation strategy (see the “Methods” section) in order to avoid overfitting. The increased SNR of the gradiometers results in a higher decoding accuracy for the best gradiometer channel (average accuracy of 81.3%) compared to the best magnetometer channel (average accuracy of 68.0%). However, both are surpassed by the best scalp-EEG channel (average accuracy of 95.6%; Fig. 1f), albeit that this might be due to the use of a classifier which was originally developed for scalp-EEG. Note that the development of a classifier tailored to OPM was out of scope for this work. With both recording modalities, the best single-channel decoding is obtained from the parieto-occipital area. Nevertheless, due to the higher spatial resolution of OPM-MEG, accurate decoding from OPMs is restricted to a smaller scalp area compared to scalp-EEG. For all three modalities, the decoding accuracy increases with an increasing number of stimulus repetitions. The often-used threshold of 70% accuracy (i.e., the minimum required to achieve reliable communication [28, 29]) is exceeded from 3, 2 and 1 stimulus repetitions for OPM magnetometers, OPM gradiometers and scalp-EEG respectively. To investigate multi-channel decoding, a greedy forward selection strategy is adopted. Briefly, one channel is iteratively added to the selected channel set until the accuracy no longer increases (for a more detailed description of this procedure, we refer the reader to the “Methods” section). When adopting this channel selection procedure within each modality, the accuracies increase considerably, and all modalities only require one stimulus repetition to surpass the decoding accuracy of 70%. The number of channels selected are 4, 6 and 4 for magnetometers (OPM), gradiometers (OPM) and scalp-EEG channels, respectively. Note that also here the OPM gradiometers yield a better performance compared to the magnetometers.

### Visual steady-state responses

**Fig. 2.**
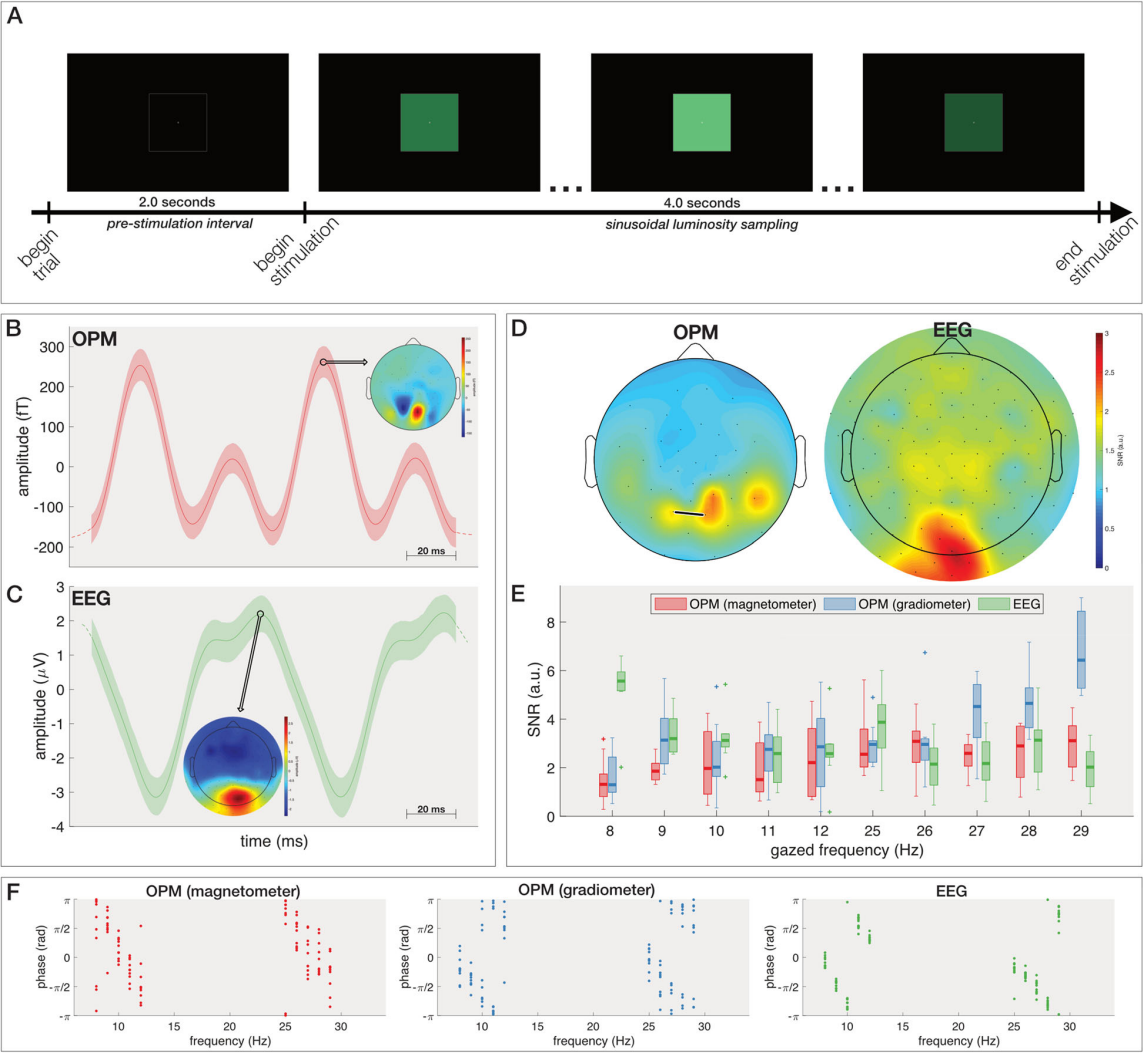
Steady-state visual evoked potential. **a** Visual rendition of one trial in the experiment. **b**,**c** Stereotypical oscillating response when gazing at a 12 Hz stimulus in the time-domain at the OPM channel **b** and EEG electrode **c** having the largest amplitude. The full line indicates the average activation and the shaded area the 95% confidence interval. The scalp plots show the activation across the scalp when the amplitude is maximal. **d** Spatial distribution of the average SNR across all stimulus frequencies for OPM (left) and scalp-EEG (right). **e** SNR in response to each gazed frequency for the OPM-magnetometer, OPM-gradiometer and scalp-EEG channel having the largest average SNR. The line segment in the boxplots indicates the median accuracy; the box stretches from the 1st to the 3rd quartile; the lines extending from the box indicate the minimum and maximum value within 1.5 times the interquartile range from the 1st and 3rd quartile, respectively, and the crosses represent outliers. **f** Phase responses are consistent across trials for all three modalities. Each dot represents one trial. Note that the negative trend in the phase response with increasing frequency is expected due to the latency of the visual system [31, 33]


While ERP/F-based BCIs are robust, the sequential nature of the stimulation does not allow one to achieve fast-paced selections. A visual BCI paradigm that allows for faster communication is based on simultaneous flickering of the selectable targets. In a second experiment, we investigated the properties of the visual steady-state responses as recorded by OPMs. To this end, we developed an interface with a single square spanning a visual angle of 9.86^∘^ centred in the subject’s foveal visual field (Fig. 2a, Additional file 5). The square flickered at different frequencies between 8 and 12 Hz (low frequency range) and between 25 and 29 Hz (high frequency range). Similar to before, the experiment was repeated twice on the same subject, once with OPM-MEG and once with scalp-EEG.

Additional file 5: Video showing several trials of the stimulation paradigm during the steady-state experiment.



Additional file 6: Video demonstrating the real-time spelling procedure for one word.


As expected, with both recording modalities, the signals obtained from the occipital scalp area exhibit periodically oscillating responses that correspond to the gazed target’s flicker frequency. To illustrate, the stereotypical oscillating response in the time-domain when gazing at a stimulus flickering at 12 Hz is shown in Fig. 2b and c for OPM and scalp-EEG, respectively. While the paradigm activates the visual cortex in both cases, a polarity reversal is visible in neighbouring occipital OPM sensors while scalp-EEG exhibits a uniform behaviour across neighbouring electrodes. Similarly, the activated regions also exhibit the highest signal-to-noise ratios. Notably, the high SNR is restricted to fewer OPM sensors compared to scalp-EEG where a larger scalp area exhibits higher SNRs (Fig. 2d), which is in line with previous reports [30]. An assessment of the SNR per gazed frequency reveals that the OPM signals are slightly more sensitive to the higher frequency range (average SNR of 2.61 and 2.58 between 25 and 29 Hz for OPM and scalp-EEG respectively) while the SNR of the scalp-EEG signals are higher in the lower frequency range (average SNRs of 2.15 and 3.51 between 8 and 12 Hz for OPM and scalp-EEG respectively) (Fig. 2e). This is especially striking when considering OPM gradiometers (average SNR of 3.77 between 25 and 29 Hz). The poorer performance at low frequencies might partially be explained by the fact that OPMs exhibit a higher internal noise at low frequencies, and, as the subject was not instructed to remain perfectly motionless, small movements relative to the remnant background magnetic field also cause magnetic artefacts in the lower frequency ranges. Finally, both the OPM and scalp-EEG signals exhibit a stable phase relationship to repeated stimulation with the same frequency (Fig. 2f), albeit that the inter-trial variability of the scalp-EEG is slightly smaller (average circular standard deviations of 0.74, 0.71 and 0.31 radians for OPM magnetometer, OPM gradiometer and scalp-EEG, respectively). Note that the negative relationship between the phase response and the gazed frequency is expected due to the latency of the human visual system [31–33].

### Accurate real-time mind-spelling with OPMs

**Fig. 3.**
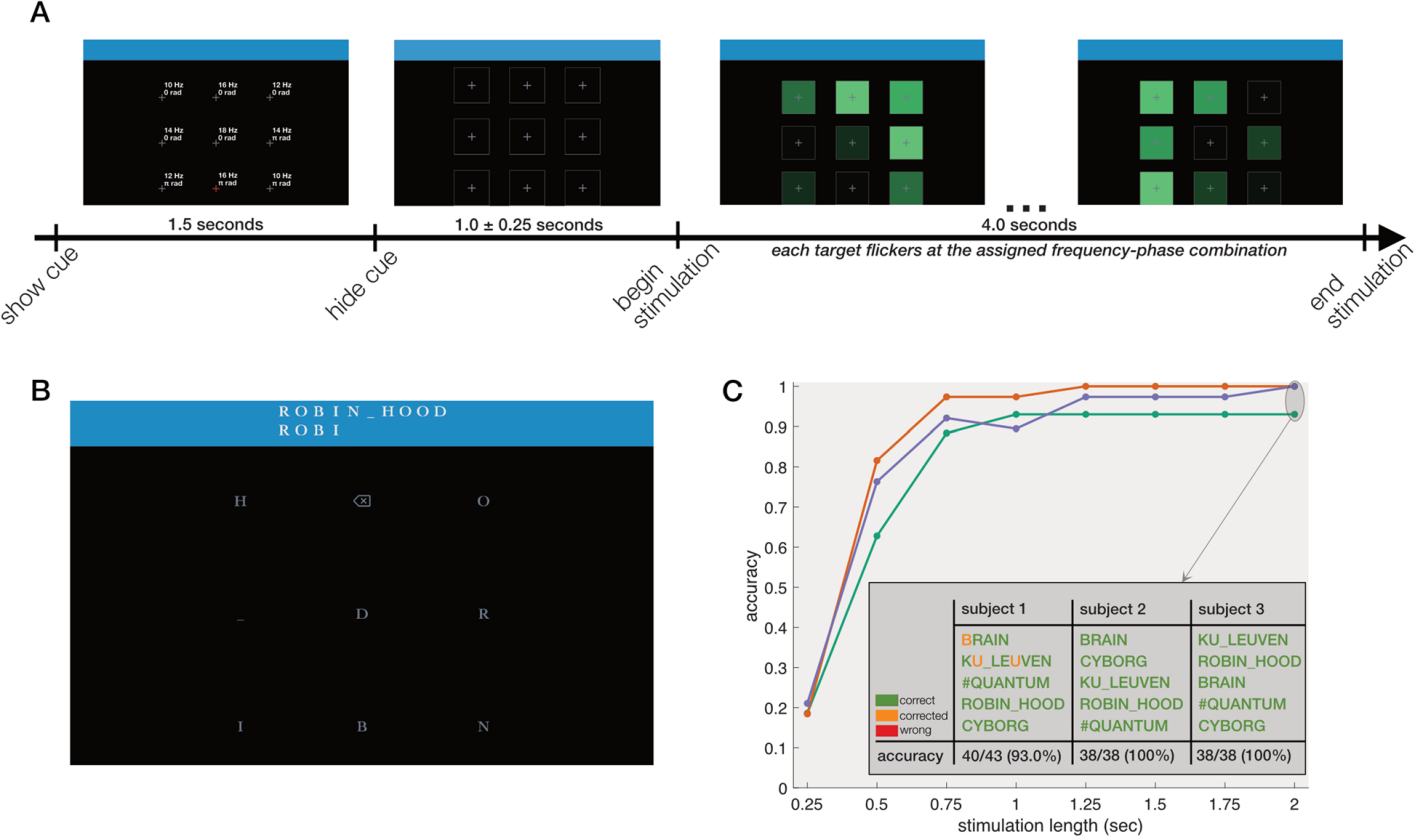
Real-time spelling. **a** Visual rendition of one trial in the training session. Note that the frequency-phase combinations shown in the first panel are for exposition purposes and were not shown during the actual experiment. **b** The interface for the spelling session showed the word to be spelled, as well as the current set of letters that had been sequentially selected by the subject in their attempt to spell out the word. Eight of the crosses have been replaced with the characters required to spell the word and one cross was replaced with a backspace icon which could be used to undo a previous selection. No cues are given and the stimulation length was reduced to 2 s. **c** Post hoc simulation of the decoding accuracies with shorter stimulation lengths. The table shows the performance of the subjects during the real-time mind-spelling session

Following the confirmation of stable single-trial measurements, we developed a real-time proof-of-concept mind-spelling interface based on visual steady-state responses. For this experiment, an interface with nine square targets was developed in which each of the targets was assigned a unique combination of frequency and phase (indicated in Fig. 3a).

Prior to the real-time spelling session, a data collection session was completed in order to acquire data to train a classifier based on spatiotemporal beamforming [34]. During the data collection session, 72 trials were completed during which the subjects were asked to visually fixate on a cued square while all squares were simultaneously flickering for 4 s (Fig. 3a). Following this session, a channel selection procedure was completed and a classification model trained. Note that while also training-free classifiers have been described in the literature, algorithms that are tuned to the specific neural activations of the current subject have shown a better accuracy [35]. The channel selection procedure selected nine (subject 1), seven (subject 2) and four (subject 3) channels to be used for decoding the gazed target. All selected channels were located over the occipital and parietal scalp area and were made up of a combination of magnetometer and gradiometer channels.

For the real-time spelling session, the interface was slightly adapted. Prior to spelling each word, the word was displayed on top of the screen and the participant was asked to spell it by consecutively gazing at the corresponding characters (Fig. 3b, Additional file 6). All words had eight or less unique characters, each of which was overlaid on one of the nine flickering squares, selected at random. One of the nine squares displayed a backspace icon that the subject could select to undo the previous selection. In total, three participants spelled five predefined words in random order. The stimulation length was further reduced from 4 to 2 s.

During the spelling session, all three participants were able to select the correct characters and had no issues completing the five words. Only three incorrect selections occurred for subject 1, all of which they were able to correct by selecting the backspace icon followed by the correct character (Fig. 3c). The other two subjects produced no incorrect selections. In total, 43 (subject 1) and 38 (subjects 2 and 3) selections were required to spell the five words, leading to final decoding accuracies during the spelling session of 93.02% (40/43, subject 1) and 100% (38/38, subjects 2 and 3). In a post hoc simulation, we evaluated the spelling performance that would have been achieved if shorter stimulation lengths had been used. This simulation shows that the stimulation length could be reduced to 750 ms without a significant loss in decoding accuracy (Fig. 3c), which would lead to a faster communication speed.

## Discussion

As future technologies are expected to interact intimately with the human brain, a practical tool for measuring reliable high-quality neural signals is needed. This study investigated the potential of a new generation MEG sensor based on optical pumping for adoption in two visual BCI paradigms. We have shown that in this context these new sensors capture neural signals with an information content that is on par with or higher than traditional scalp-EEG, and we have demonstrated reliable single-trial decoding using OPM-MEG in a proof-of-concept, real-time spelling application.

### Magnetic Brain-Computer Interfacing

The vast majority of BCI work has focussed on non-invasive electrophysiology in the form of scalp-EEG. While much neural information is lost by the conduction through the scalp, the non-invasive nature of scalp-EEG renders it more practical than the use of invasive neural implants (e.g. ECoG [6], depth probes [8], micro-electrode arrays [7] or flexible electrode threads [36]) and allows for a wider adoption of neural interfaces by the general population. However, the spatial blurring of cortical activations over a large scalp area limits its potential for next-generation neurotechnologies as it becomes nearly impossible to differentiate signals that originate from neural sources that are anatomically close to one another (e.g. decoding of individual finger movements [37]). In contrast to the electrical potentials, the neural activity contained within the complementary magnetic domain is less distorted spatially by the inhomogeneous conductivity profile of the head, and its measurement allows neural activity to be captured with a higher spatial precision [30, 38]. In agreement with this, we found that the N200 ERP in response to the motion-onset paradigm recorded by scalp-EEG indeed dominates large portions of the scalp topography while the M200 OPM-MEG response is more local to the active neural dipole, evidenced by the polarity shift across OPM sensors. Moreover, this polarity reversal can be exploited by gradiometer channels to obtain signals with a higher SNR of the component-of-interest. Successful exploitation of the increased SNR and spatial accuracy will likely result in neural interface applications with more advanced features.

While also traditional SQUID-based MEG systems surpass scalp-EEG in terms of spatial resolution and SNR, the bulky and costly recording hardware hampers the adoption of MEG-based neural interfaces. Indeed, only a limited number of reports on MEG-based BCI are available in the scientific literature, and the majority of these studies have focused on the decoding of (imagined) movements of limbs [39–43] or mental tasks [44] as these paradigms allow the subject’s head to remain confined within the MEG helmet. This implies that many paradigms that are described in EEG-studies have no counterpart in MEG and that considerably fewer signal features or models for decoding the subject’s intention have been described. Additionally, unlike scalp-EEG, OPM-MEG does not require an extensive setup time as there is no need for the application of conductive gel. This allows a faster turn-around time as OPMs do not require extensive cleaning which makes the system in practice immediately available for the next participant. Finally, unlike traditional SQUID-based MEG, OPMs are less prone to head movement artefacts. Given the right countermeasures (e.g. dynamic nulling, motion tracking), even paradigms in which subjects perform active movements are feasible [25]. In the context of neural interfaces, this would allow the adoption of paradigms or therapies (eg, neuro-steered rehabilitation programmes [45, 46]) to extend beyond hand or arm movements [47] or mental tasks, and even opens up possibilities to include patients that suffer from involuntary muscular activity, such as in spastic cerebral palsy. Given the similarities between the OPM-MEG and scalp-EEG setups in terms of movement restrictions, most of the paradigms adopted in EEG studies can easily be translated to OPM-MEG.

In this study, we adopted neural decoders that were previously developed for scalp-EEG [48] as the development of novel features or decoders was considered out of scope for this study. While this classifier has shown state-of-the-art performance on scalp-EEG for the considered paradigms, it has also been shown to be less suited for decoding intracranial neural activations [49], likely due to the more narrow spatial activation of both the component-of-interest as well as potential noise sources. We believe it is therefore likely that a model that accounts for the magnetic properties of the signal would further improve decoding performance. For prolonged use of a BCI system, it would be beneficial to develop decoders in source space rather than scalp space; this would exploit the well characterised interference rejection properties of source localisation algorithms and result in decoding using a higher SNR signal. Decoding in source space could furthermore take advantage of more detailed spatial characteristics elicited by the paradigm (e.g. activations according to the retinotopic map) to complement the temporal signal morphology.

### Practical considerations

It is worth mentioning that, at the time of writing, OPM-MEG is a new technique that is under active development. As evidenced by the larger inter-trial variability in phase responses to the visual flickers and larger confidence interval of the M200 latency, the inter-trial stability of OPM-MEG still seems to trail those of scalp-EEG. For BCI paradigms that rely on time-locked neural responses, this temporal variability could results in a reduced performance. Phase-based SSVEP paradigms, for example, could struggle to accurately discriminate between targets that are encoded with the same frequency but different phase [50], and with ERF-based paradigms, a larger variability in latencies could reduce the temporal precision of ERF components which might result in less pronounced interpretations. Our demonstration, however, shows that the current implementation of OPM-MEG already allows for reliable decoding. Furthermore, as an increasing number of research groups are acquiring OPM hardware, we believe the OPM-MEG technology will still experience tremendous strides in terms of signal quality and stability, spatial accuracy, analysis methodologies and MEG-applications. In addition, the development of standardised procedures for OPM-MEG (e.g. scalp locations, similar to the 10–20 system used in scalp-EEG studies) will benefit multi-centre collaborations and cross-study generalisations. Furthermore, given their compact size and flexible deployment capabilities, OPMs can be incorporated in specialised equipment for specific purposes. Examples include a cylindrical tube which has the OPMs built into the headrest [27], mouth magnetoencephalography for capturing hippocampal activity [51] or sensor belts for magnetocardiography [52, 53]).

To date, successful OPM-MEG requires the recording to be carried out inside a magnetically shielded room to mitigate the effects of everyday-life magnetic sources (e.g. the earth’s magnetic field or electrical devices). Furthermore, to obtain maximal sensitivity to the magnetic disturbances that originate from neural activity and to reduce the influence of movement artefacts, the remnant background magnetic fields in the shielded room should be cancelled using an active nulling technique [54–56].

With these considerations in mind, we envisage that commercial OPMs will initially be beneficial in clinical contexts as hospitals typically have the resources to invest in a magnetically shielded room and OPM system. Clinically-oriented neuro-technologies (e.g. neuro-steered cognitive training [57], rehabilitation therapies [15] or auditory attention assessments [58]) can then easily be deployed, as well as offering new diagnostic procedures, such as foetal magnetocardiography [52, 59], localisation of epileptic foci [60] and objective assessments of language disorders [61].

### Limitations

In the current study, only a limited group of participants was recruited. We are, however, confident that the reported results extrapolate to a larger population given that the reported paradigms have been widely adopted in the scientific literature [62–64]. The visual steady-state paradigm, in particular, is the most adopted paradigm in visual BCI studies and has been successful in numerous applications (e.g. spelling [65–67], wheelchair control [68], games [69]) and across all ages of the population [70].

## Conclusions

In support of the proliferation of future neural interface technologies, a tool for reliable non-invasive acquisition of high-quality neural signals is required. In this work, we have demonstrated the potential of a new generation MEG sensor based on optical pumping. Using ‘out-of-the-box’ decoders, OPM-MEG has proven to allow robust single-trial decoding which was demonstrated by the accurate control of a real-time ‘mind-spelling’ application. Given the high spatiotemporal resolution, flexible deployment capabilities and low maintenance of the hardware, we believe OPM will play an important role in the further development of neural interfaces.

## Methods

### OPM-MEG system

All experiments were performed using a whole-head multi-channel OPM-MEG system containing 48 second-generation, zero-field magnetometers manufactured by QuSpin Inc. (CO, USA). Each sensor is a self-contained unit, of dimensions 12.4 ×16.6 × 24.4 mm^3^, containing a Rb-87 gas vapour within a heated glass cell, a laser for optical pumping, and on-board electromagnetic coils for controlling the local magnetic field within the cell. Precisely how this device measures magnetic field has been dealt with in previous reports [20] and this information will not be repeated here. The OPMs were mounted on the participant’s head using a rigid, 3D-printed helmet [23] and each sensor was connected via a 60-cm lightweight (3.3 g/m) flex cable, to a backpack. Thicker cables were then taken from the backpack to the control electronics. Analogue output signals were fed from the OPM electronics to a National Instruments digital acquisition system (DAQ). Although OPMs can measure two orthogonal components of the magnetic field, we only measured the component of the magnetic field that was normal to the scalp surface in the experiments reported here. Importantly, prior to the start of any experiment, all OPMs were calibrated using a manufacturer established procedure. In brief, on-board-sensor coils were energised to produce a known field within the cell, the output of the sensor was then measured and calibrated to ensure a response of 2.7 V/T.

The system was operated within a magnetically shielded room (MSR) designed and built specifically for OPM operation (MuRoom, Magnetic Shields Limited, Kent, UK). This MSR, which comprises two mu-metal layers and a single copper layer, was equipped with degaussing coils [71], effectively reducing the background static magnetic field to ∼1.5 nT, with field gradients of less than 2 nT/m. The operational dynamic range of the QuSpin zero-field magnetometers (which we define here as the maximum change in field before gain errors become >5%) is ∼1.5 nT [25]. In an MSR with a background field of 30 nT, this would mean a head rotation of around 3^∘^ is enough to generate a 1.5-nT field change, which would, in turn, cause a significant (>5%) change in gain of the OPM. In our MSR, an OPM can be rotated through 360^∘^ about any axis and still maintain gain error within 5%.

Even though OPMs remain operational in the low background field inside our MSR, head movement within this field still generates artefactual signals which can distort measured brain activity. For this reason, the background field and gradients were further controlled using a set of bi-planar coils placed on either side of the participant [54, 55]. These coils, which are wound on two 1.6-m square planes separated by a 1.5-m gap in which the participant is placed, generate three orthogonal magnetic fields and four of the five independent linear gradients within a (hypothetical) 40 cm cube inside which the participant’s head is positioned. A reference array, placed behind the participant, then measures the background field/gradient and currents are applied to the bi-planar coils to cancel this remnant field. This takes the background field from 1.5 nT to ∼0.5 nT, which enables a threefold improvement in suppression of movement artefacts.

**Fig. 4.**
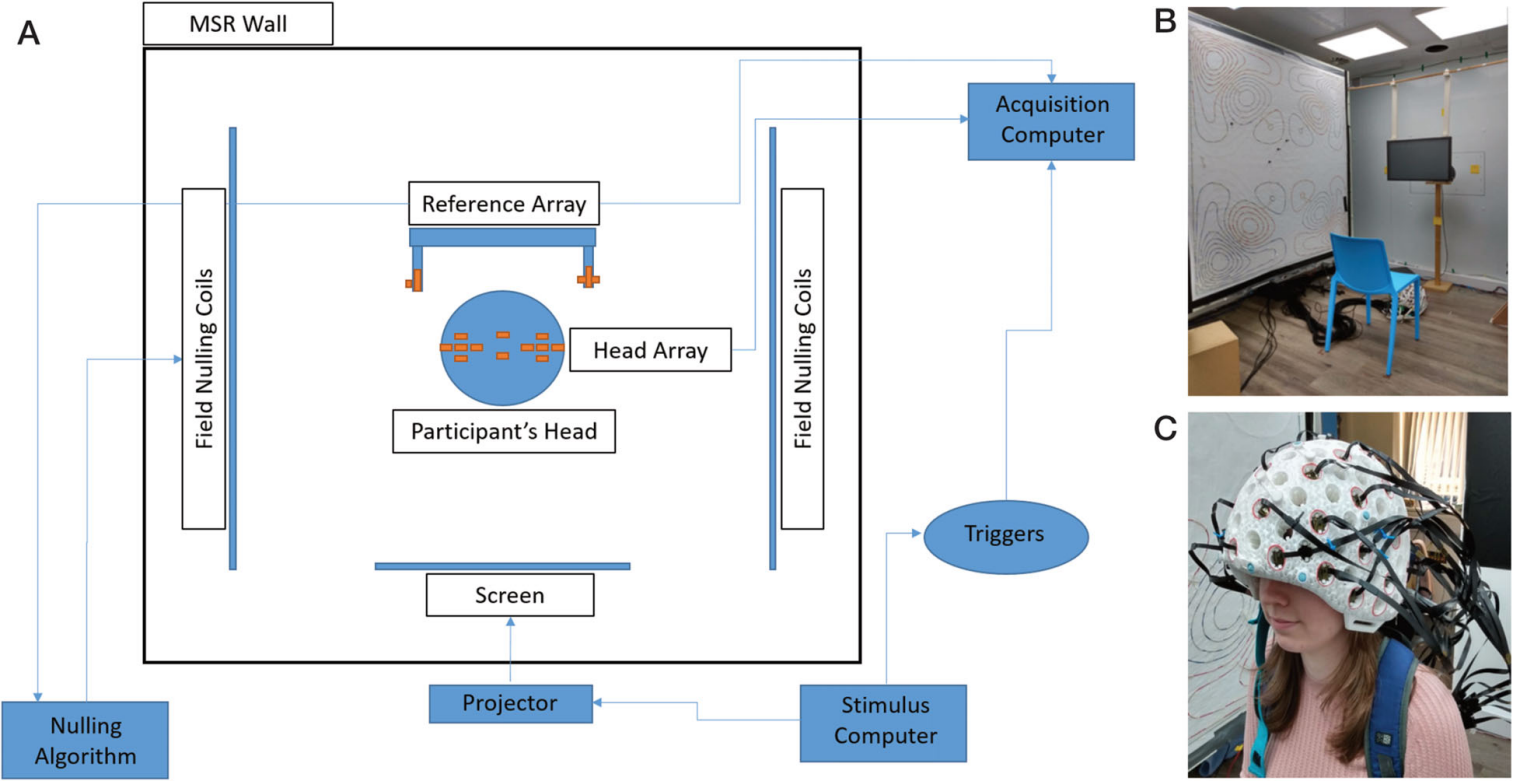
**a** Schematic diagram of the full OPM setup. Note that during the real-time spelling experiment, the stimulus and acquisition computer were the same device. **b** View inside the magnetically shielded room. **c** Example of the rigid helmet with OPM sensors at different scalp locations

A schematic diagram of the system is shown in Fig. 4. The participants sat on a non-magnetic chair placed in the centre of the MSR between the bi-planar coils. Note that all control electronics are kept outside the MSR in order to minimise the effect of magnetic interference on the MEG measurements.

### Motion-onset visual evoked potential

#### Experimental procedure

The experimental interface consisted of nine crosses arranged in a 3-by-3 matrix design, each one spanning a visual angle of 0.86^∘^ with an inter-cross distance of 5.17^∘^. At the beginning of each trial, one of the crosses was cued by changing its colour from grey to red. The subject was instructed to redirect his gaze to this cross, maintain visual focus and mentally count the number of times this cross would expand during the duration of the subsequent trial. 1000 ms following the onset of the cue, the cued cross would regain its grey colour and 1000 ± 75 ms later the stimulation starts. The stimulation consisted of the expansion to 4.3^∘^ followed by a contraction to the original size of one cross in a time span of 150 ms. This was repeated in pseudorandom order (block design) with an inter-stimulus interval of 150 ± 75 ms such that each of the nine crosses was stimulated 5 times. At the end of a trial, the next cue was presented.

One male subject (28 years old, right handed) with normal visual acuity participated in the experiment. He repeated this experiment twice, one with OPM and once with EEG. For the OPM-session, the subject was seated in a magnetically shielded room (Magnetic Shield Limited, UK) and the experimental interface was presented at a refresh rate of 60 Hz using an GT1080Darbee projector (Optoma, UK) located outside the magnetically shielded room and projected on a projection screen approximately 80 cm in front of the subject. The optically pumped magnetometers (QuSpin, USA) were placed in a 3D-printed rigid helmet evenly distributed across the scalp [23]. Prior to the experiment, the magnetically shielded room was degaussed to remove residual magnetic charges in the room. The active nulling was static and thus did not change during the entire duration of the experiment.

For the EEG-session, the subject was seated in a comfortable desk chair in a sound-proof airconditioned room and the experimental interface was presented using a true 120 Hz monitor (Viewpixx, Canada). 128 active Ag/AgCl electrodes were evenly distributed over the scalp at locations according to the international 10/20 system, with ground and reference electrodes located at Fpz and FCz respectively. The signals were recorded at a sampling rate of 1000 Hz using a Neuroscan SynampsRT device (Compumedics Europe, Germany). Conductive gel was applied at each electrode to increase signal strength and reduce noise. All impedances were kept below 1 k *Ω*.

#### Data analysis

**Preprocessing** Prior to analysis, the EEG data was re-referenced to the average of the mastoid signals. As MEG is reference-free, no additional preprocessing was done on the OPM signals prior to analysis.

**ERP/F** To extract the ERP/F, both the OPM and (re-referenced) EEG signals were first filtered between 0.5 and 15 Hz using a 4th-order zero-phase Butterworth filter. Next, epochs were extracted from 200 ms before to 600 ms after each stimulus onset and labelled with the cued and stimulated cross. Finally, each epoch was baselined by subtracting the average activity during the corresponding 200 ms pre-onset window. All epochs for which the stimulated target corresponded to the cued target were averaged to obtain the ‘target ERP’ while the others were averaged to obtain the ‘non-target ERP’. The 95% confidence interval was obtained using a bias-corrected and accelerated bootstrapping procedure [72] with 1000 bootstrap samples (Matlab’s *bootci* function). The latency of the N/M200 and P/M300 ERP/F was given by the timepoint at which the amplitude reached its maximal level across all channels. The 95% confidence interval was obtained using a bias-corrected and accelerated bootstrapping procedure [72] with 1000 bootstrap samples (Matlab’s *bootci* function).

**SNR** The signal-to-noise ratio of the N/M200 and P/M300 components of the target ERP/Fs was extracted using the procedure detailed in [73]. For both components, the window-of-interest was set to a 100 ms window centred at their respective peak-latency. The 95% confidence interval was calculated using a bootstrapping procedure with 1000 samples and the percentile approach.

**Decoding the gazed target** To determine the decoding accuracy, a fivefold cross-validation strategy was followed. In each iteration, fourfolds were used to train a state-of-the-art classifier (see further) while the remaining fold was used to assess the decoding accuracy. All predictions were accumulated in a confusion matrix and the reported accuracy was given as the ratio of the correct predictions (i.e. sum of diagonal of the confusion matrix) divided by the total number of predictions.

As a classifier, we chose a model based on spatiotemporal beamforming as it has shown state-of-the-art performance for ERP-based decoding of gazed targets [48, 74]. The spatiotemporal beamformer estimates the contribution of an a-priori defined activation pattern to the current segment of data. As activation pattern $\mathbf {a} \in \mathbb {R}^{1 \times mn}$, where *m* is the number of channels and *n* the number of samples, the averaged target ERP/F obtained from the training epochs was used and vectorised. The covariance matrix $\Sigma \in \mathbb {R}^{mn \times mn}$ was calculated using all vectorised training epochs and regularised with a regularisation constant *α*=0.95: $\hat \Sigma = \alpha \Sigma + (1-\alpha)I$, where *I* is the identity matrix. Using the constraint **aw**=1, the linearly-constrained minimum-variance (LCMV) beamformer can then be calculated as follows [75]:

1 $$ \mathbf{w} = \frac{\mathbf{a}\hat\Sigma^{-1}}{\mathbf{a}\hat\Sigma^{-1}\mathbf{a}^{\intercal}},  $$

where $\hat \Sigma ^{-1}$ is the pseudo-inverse of $\hat \Sigma $. Given a set of epochs with their respective labels, a prediction was made by obtaining the average epoch per unique label and applying the beamformer to each vectorised average response. The label having the maximal beamformer output was indicated as winner. For a more detailed description of the classification scheme, we refer the reader to previous works [48, 74].

The simulation was repeated for an increasing number of stimulus repetitions (from one to five) and for each individual EEG and OPM channel, as well as for all OPM-gradiometer channels to assess single-channel decoding accuracy. For multi-channel decoding, a greedy forward channel selection strategy was used which terminated when the average decoding accuracy from one to five stimulus repetitions of subsequent iterations no longer improved or when a decoding accuracy of 100% was reached. While other channel selection strategies have been suggested [76], greedy forward selection was chosen due to its intuitive design and straightforward implementation.

### Steady-state visual evoked potential

#### Experimental procedure

The experimental interface consisted of one square spanning a visual angle of 9.86^∘^. In the centre of the square, a fixation cross was presented and the subject was asked to visually focus on this cross during the duration of the experiment. During each trial, the square was flickered for 4 s at a given frequency *f* by sinusoidally modulating its luminosity. The inter-trial interval was 1.75 ± 0.25 s. Each of the integer frequencies from 8 and 12 Hz and from 25 to 29 Hz were presented 10 times in a pseudorandom order (block design), leading to a total of 100 trials. In between blocks, a 10-s break was given to the subject.

One male subject (28 years old, right handed) with normal visual acuity participated in the experiment. He repeated this experiment twice, once with OPM-MEG and once with EEG. OPM and scalp-EEG data were acquired in the same setting as described in previous section with one exception: in order to avoid sampling artefacts in the sinusoidal luminosity profile, the projector used in the OPM experiment was set to a refresh rate of 120 Hz.

#### Data analysis

**Preprocessing** Prior to analysis, the EEG data was re-referenced to the average of the mastoid signals. As MEG is reference-free, no additional preprocessing was done on the OPM signals prior to analysis. The continuous recordings were then cut into 4-s epochs locked to the onset of each trial. Each epoch was labelled with the frequency that was presented during the corresponding trial.

**Time-domain** Prior to the time-domain analysis, the epochs were filtered between 4 and 40 Hz using a fourth-order zero-phase Butterworth filter to isolate the fundamental component. Next, for each stimulation frequency *f*, all epochs labelled with frequency *f* were cut into (50%) overlapping segments whose length equals two periods of the corresponding gazed frequency *f*. All segments were then averaged to obtain the stereotypical response in the time-domain. The 95% confidence interval was extracted using an accelerated and bias-corrected bootstrapping procedure with 1000 bootstrapped samples [72]. The scalp plots render the amplitude of the time-domain activation across the sensors at the time point for which the maximal value across all sensors was reached.

**SNR** The signal-to-noise ratio for each epoch was obtained from the frequency spectrum. First, the frequency spectrum for each epoch was obtained using the Fourier transform and the amplitude at the gazed frequency was extracted. Next, this amplitude was normalised relative to the average spectral amplitude of the six neighbouring samples on each side of the gazed frequency. The scalp plots in Fig. 2d and e visualise the distribution of the average SNR across all frequencies.

**Phase response** The phase response of each epoch was obtained by applying the Fourier transform followed by the extraction of the phase using Matlab’s *angle* function. For each unique gazed frequency, the circular standard deviation was obtained from the corresponding epochs using the CircStat toolbox [77].

### Real-time mind-spelling

#### Experimental procedure

The experimental interface consisted of nine squares in a 3 ×3 matrix design presented on a projection screen using an GT1080Darbee projector (Optoma, UK) operating at a refresh rate of 60 Hz. Each square spanned a visual angle of 4.3^∘^ in the horizontal and vertical dimensions. The inter-square distance was 2.7^∘^, horizontally and 1.7^∘^ vertically. During the training session, each square was overlaid with a fixation cross that spanned a visual angle of 0.86^∘^. Each of the nine squares was assigned a unique frequency-phase combination, as shown in Fig. 3a. Three subjects (1 female, aged 40, 22 and 26 years old, all right handed) with normal visual acuity participated in the experiment. Subjects were seated in a magnetically shielded room (Magnetic Shield Limited, UK) at approximately 80 cm from the projection screen. The optically pumped magnetometers (QuSpin, USA) were placed in a specially designed helmet [23]. The sensors were uniformly distributed across the scalp. Unlike the previous two experiments, the active magnetic shielding was dynamic and adapted to small changes in the magnetic field experienced by the OPMs throughout the entire experimental session by using the sensitive outputs of three of the four reference magnetometers (one measurement for each cartesian component of the magnetic field) as inputs to a high-speed proportional integral controller [55].

#### Training session

Prior to the real-time spelling session, the participant first completed a training session, aimed at collecting data used to train a classifier (see further). A trial in the training session started with a visual cue during which one of the nine fixation crosses adopted a red colour, and the subject was asked to redirect his gaze to this target. After a jittered interval of 1.0 ±0.25 s, the cue was removed and the nine targets started flickering at their assigned frequency-phase combinations, achieved by adopting a sinusoidal luminosity profile [78]. After 4 s, the flickering stopped and the trial was ended. Each target was cued 8 times in pseudorandom order (block design), leading to a total of 72 4-s trials. The total training session lasted approximately 8 minutes. Data was collected continuously throughout the duration of the training session.

#### Data processing

The raw OPM data collected during the training sessions was filtered between 4 and 40 Hz using a fourth-order zero-phase Butterworth filter, cut into 4-s epochs locked to the onset of each trial, downsampled to 150 Hz, and labelled with the frequency-phase combination of the corresponding gazed target.

#### Decoder

From the preprocessed data, a classification pipeline based on spatiotemporal beamforming was trained. For each of the nine unique frequency-phase combinations, a spatiotemporal beamformer [34, 49, 67] was constructed that estimates the contribution of the corresponding frequency-phase combination into the current segment of data. The beamformer for target *i*∈[1..9] was calculated by obtaining an activation pattern $\mathbf {a}_{i} \in \mathbb {R}^{1 \times mn}$ and a regularised covariance matrix $\hat \Sigma _{i} \in \mathbb {R}^{mn \times mn}$ for target *i*∈[1..9], where *m* is the number of channels and *n* the number of samples in 2 periods. First, all epochs during which target *i* was cued were then cut into (50%) overlapping segments whose length equals two periods of the stimulation frequency *f*_*i*_ of target *i*. The activation pattern was then obtained as the average segment $\mathbf {A}_{i} \in \mathbb {R}^{m \times n}$ and vectorised to obtain $\mathbf {a}_{i} \in \mathbb {R}^{1 \times mn}$. The covariance matrix *Σ*_*i*_ was estimated from all available epochs by extracting segments using the procedure above. Note that also epochs not corresponding to the stimulation frequency under consideration are cut into segments of length *n*. A regularisation constant of 0.95 was adopted in the calculation of the covariance matrix, similar to before. Using the constraint **a**_*i*_**w**_*i*_=1, the linearly-constrained minimum-variance (LCMV) beamformer for target *i* can then be calculated as follows [75]:

2 $$ \mathbf{w}_{i} = \frac{\mathbf{a}_{i}\hat\Sigma_{i}^{-1}}{\mathbf{a}_{i}\hat\Sigma_{i}^{-1}\mathbf{a}_{i}^{\intercal}},  $$

where $\Sigma _{i}^{-1}$ is the pseudo-inverse of *Σ*_*i*_.

Given an epoch, a prediction was made by iteratively extracting the average segment for each target and applying the corresponding beamformer. The winning frequency-phase combination corresponded to the beamformer with maximal output. For a more detailed description of the classification scheme, we refer the reader to previous works [34, 48, 49, 67].

#### Channel selection

In order to reduce the dimensionality of the decoding model, a greedy forward channel selection strategy was adopted. Starting with an empty set, every iteration defines candidate sets as the currently selected channels extended with each of the non-selected channels. The channel that increases the decoding performance the most is added to the final set. Candidate channel sets were scored using a fourfold cross-validation on the training epochs. While this heuristic approach is not guaranteed to find the optimal channel set, its simplicity has made it a widely adopted approach [76]. Of the 48 OPM channels recorded during the entire experiment, we manually selected 24 OPM sensors and 45 gradiometers located over the parieto-occipital scalp area prior to the experiment in order to speed up the channel selection procedure. As this reduces the number of candidate channel sets, fewer models need to be trained and evaluated during the channel selection. In total, the channel selection procedure lasted about two minutes, during which the subject was asked to relax while waiting for the spelling session to start.

#### Real-time spelling

Following the training session, channel selection and classifier training, the real-time spelling session was initiated. Subjects were asked to spell five predefined words. A block began with the presentation of the word-to-spell on top of the interface and replacing the fixation crosses by the characters that are required to spell the current word, distributed in a random fashion. Additionally, one of the fixation crosses was replaced with a backspace icon the subject could use to undo previous selections. In case the word only required six or less different characters, the remaining fixation crosses were replaced with other randomly chosen characters. All characters spanned a visual angle similar to the fixation crosses. Following a 20-s habituation period, the spelling procedure started. A 2-s flickering stimulation was presented and the corresponding brain responses were obtained in real-time from the OPM sensors. The recorded data was then filtered between 4 and 40 Hz using a fourth-order zero-phase Butterworth filter and submitted to the classification pipeline for decoding the gazed target. For each of the nine beamformers, the 2-s epoch was cut into segments of the corresponding frequency, and the segments corresponding to the initial 150 ms were removed. The remaining segments were averaged and applied to the trained beamformer to obtain an estimate (i.e. score) of the presence of the corresponding frequency-phase combination. The beamformer resulting in the highest score was indicated as winner and the character with the corresponding frequency-phase combination was highlighted in yellow. The character by character selection was also displayed on top of the screen, under the word to be spelled. This procedure was repeated until the spelled word contained as many characters as the target word.

#### Post hoc analysis

While the real-time session was performed using a 2-s stimulation, in a post hoc simulation the decoding accuracies with shorter stimulation lengths were estimated. To this end, all epochs from the spelling session were shortened by retaining the initial *n* seconds and presented to the classifier trained on the training set. The predictions were then compared to the actual gazed characters and reported in Fig. 3c. This procedure was repeated for increasing signal lengths *n* from 250 ms to 2 s in steps of 250 ms.

## Supplementary Information

**Additional file 4** Spatial distribution of the signal-to-noise ratio for the N/M200 (subfigures A and B) and P/M300 (subfigures C and D) ERP/F in response to the motion-onset paradigm. The full line in subfigures A and C indicates the gradiometer channel exhibiting the largest SNR.

## Data Availability

The datasets generated during and/or analysed during the current study are available from the corresponding author on reasonable request. The MATLAB (version 2019a) code used to analyse the current study is available from the corresponding author on reasonable request.

## Abbreviations

BCI: Brain-computer interface
EEG: Electroencephalography
MEG: Magnetoencephalography
SQUID: Superconducting quantum interference device
OPM: Optically pumped magnetometer
ECoG: Electrocorticography
ERP: Event-related potential
ERF: Event-related field
SSVEP: Steady-state visual evoked potential
SNR: Signal-to-noise ratio
MSR: Magnetically shielded room
