# Supplementary figures and images for: Practical real-time MEG-based neural interfacing with optically pumped magnetometers
Source: BMC Biol. 2021 Aug 10;19:158. doi: 10.1186/s12915-021-01073-6 (PMC8356471; doi:10.1186/s12915-021-01073-6)

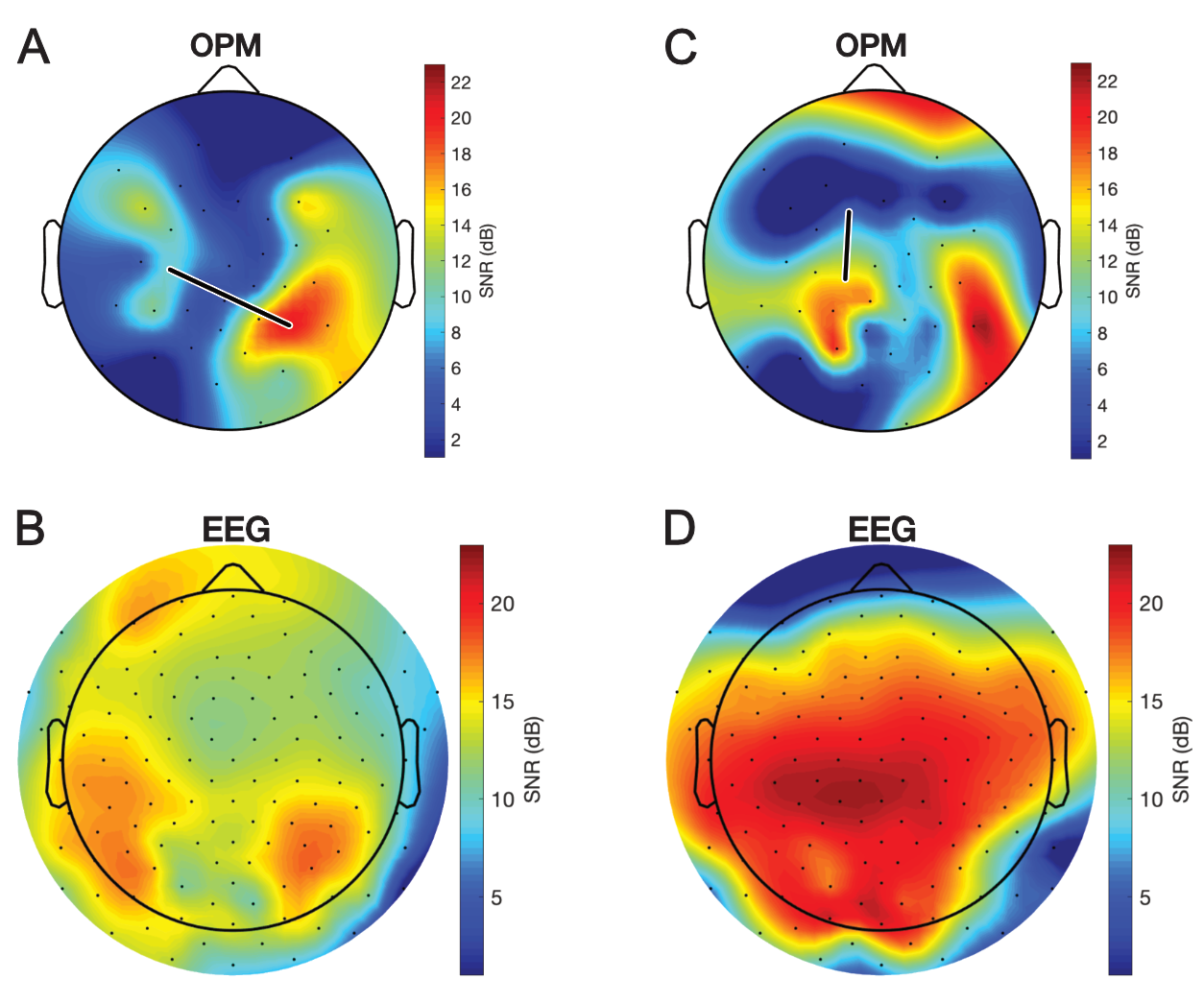

Supplement: Supplementary file 6 — Additional file 4 Spatial distribution of the signal-to-noise ratio for the N/M200 (subfigures A and B) and P/M300 (subfigures C and D) ERP/F in response to the motion-onset paradigm. The full line in subfigures A and C indicates the gradiometer channel exhibiting the largest SNR. [file 12915_2021_1073_MOESM4_ESM.tif]
